# Spatial linear transformer and temporal convolution network for traffic flow prediction

**DOI:** 10.1038/s41598-024-54114-9

**Published:** 2024-02-19

**Authors:** Zhibo Xing, Mingxia Huang, Wentao Li, Dan Peng

**Affiliations:** School of Transportation and Geomatics Engineering, Shenyang Jianzhu University, Shenyang, 110168 Liaoning China

**Keywords:** Traffic forecasting, Deep learning, Spatial linear transformer, Bidirectional temporal convolution network, Dynamic global spatial dependency, Engineering, Civil engineering

## Abstract

Accurately obtaining accurate information about the future traffic flow of all roads in the transportation network is essential for traffic management and control applications. In order to address the challenges of acquiring dynamic global spatial correlations between transportation links and modeling time dependencies in multi-step prediction, we propose a spatial linear transformer and temporal convolution network (SLTTCN). The model is using spatial linear transformers to aggregate the spatial information of the traffic flow, and bidirectional temporal convolution network to capture the temporal dependency of the traffic flow. The spatial linear transformer effectively reduces the complexity of data calculation and storage while capturing spatial dependence, and the time convolutional network with bidirectional and gate fusion mechanisms avoids the problems of gradient vanishing and high computational cost caused by long time intervals during model training. We conducted extensive experiments using two publicly available large-scale traffic data sets and compared SLTTCN with other baselines. Numerical results show that SLTTCN achieves the best predictive performance in various error measurements. We also performed attention visualization analysis on the spatial linear transformer, verifying its effectiveness in capturing dynamic global spatial dependency.

## Introduction

**Figure 1:**
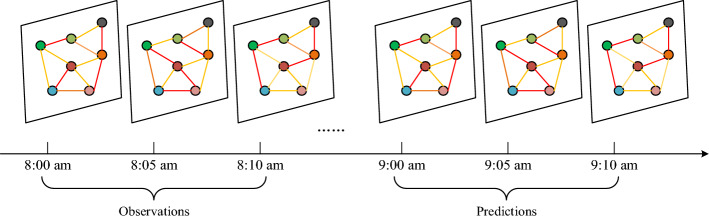
The observations and predictions for traffic states, where the different color node represent the different area and different lines connecting the area represent the traffic states evolving with time.

Traffic state prediction can be regarded as the problem of spatial–temporal prediction, the traffic states (volumes, speeds, occupancy, etc.) are recorded at a fixed time with corresponding locations which are distributed in continuous space. As is shown in Fig. 1, it is evident that, on the one hand, the predictions of future traffic states are impacted by the observations of historical traffic states with respect to temporal factors, on the other hand, the traffic states of currents area are influenced by its neighbor areas with respect to spatial factors. Therefore, we need to mining the temporal and spatial relationship of traffic data to help us predict the traffic states efficiently.

Nowadays, the transportation technology brings convenience for collecting traffic data. The traffic device detects the traffic data in real time with very short time interval so the characters of the traffic data are non-linear and complex. Therefore, it is a challenge to discover the inherent temporal-spatial relationship and predict the traffic state accurately. After decades of studies and practices, various approaches of traffic prediction have come into being. For statistical approach, models of time series analyses, especially the autoregressive integrated moving average (ARIMA), are widely used in traffic prediction^1^ firstly applied the ARIMA model for incident detection in univariate single-point settings, then a lot of variations^2,3^ come into being. For machine learning approach, the classical models including the k-nearest neighbor (KNN)^4^ and support vector machine (SVM)^5^. These approaches can model more complex traffic data but they require the careful feature engineering.

Recent years, the deep learning approaches, as a kind of end-to-end learning mode, can handle nonlinear high-dimensional problems, which has attracted interest of researchers. Furthermore, the graph neural network (GNN) as one of the deep learning fields, has been proposed for temporal-spatial modeling^6^. Vividly, the sensors in road network are like the nodes^7,8^ in graph, and the connectivity between sensors are like the edges between nodes with the weights. In recent years, a popular and effective analysis has emerged, that is combing the GNN with the sequence learning model. For instance^9,10^, integrate the graph convolution network (GCN) with recurrent network (RNN) to introduce the inherent road network topology into time series model, so their performance apparently improves. With the rise of attention mechanisms^11^ in various fields, many studies^12,13^ have combined GNN with transformers to model spatio-temporal dependencies. It can capture the correlation coefficients of multi-dimensional features of raw data and obtain more accurate prediction results. However, the above studies are having two issues to overcome in temporal and spatial dependency modeling.

For spatial dependency, the problem is that they explore spatial dependency based on the assumption of a predefined graph structure which is impacted directly by their spatial connectivity. But in some situation the spatial connectivity cannot correctly reflect the real dependency between two nodes. To solve this problem, the graph attention network (GAT) theory came into being. The GAT^14,15^ focuses on construct the dynamic spatial dependency by compute the spatial dependency from the traffic state of object node to the relational node and it becomes a popular research direction.

For temporal dependency, the problem is that despite the wide usage of RNN and its variants^16,17^ in capturing temporal dependencies, they still suffer from drawbacks such as time-consuming computations, complex gating mechanisms, and slow response to dynamic changes. In addition, it inconvenient to deal with the long-term temporal dependency by stacking CNN layers whose receptive field size grows linearly with the number of layers increasing^18,19^. The problem faced by the transformer structure is that as the time series grows, the computational complexity of its internal self-attention mechanism increases significantly, which greatly affects the performance of the transformer in handling long sequence data.

Two address the above two limitations, we proposed a new framework called Spatial Linear Transformer with Temporal Convolution Network (SLTTCN). With regard to spatial dependency, we proposed a spatial linear transformer network (SLTN) which aims at dynamically capture the spatial dependency by taking the real-time traffic state, connectivity among nodes and traffic flow directions into consideration. The classical transformer^20^ has the disadvantage of high memory and computational complexity. Therefore, to make the SLTN works efficiently, we optimize the original form of self-attention mechanism into a linear form to reduce the resource demand of SLTN. With regard to temporal dependency, inspired by the temporal convolution network (TCN)^21^, we capture temporal dependency stack the by dilated casual convolution layers, and the receptive field size grows exponentially with the number of stack layers increasing. In additional, we introduce a bidirectional and gate-fusion mechanism to TCN, that is mining the temporal dependency from past to future and from future to past, then fuse the two kinds of dependency to express the complex temporal dependency better. The main contributions are summarized as follows:

- We construct a novel framework named Spatial Linear Transformer with Temporal Convolution Network (SLTTCN) to dynamically model the temporal and spatial dependency of traffic flow.
- We propose the spatial linear transformer network (SLTN) to dynamically capture the spatial dependency, and for improving the efficiency of network, we change the structure of SLTN to reduce the memory and computational complexity.
- We propose the temporal convolution network with bidirectional and gate fusion mechanism to capture the temporal dependency to take the advantage of steady gradient, parallel computing and simple structure.

The structure of the paper is as follows: In section "Introduction" we introduce the reason for traffic prediction, the issues for modeling the temporal and spatial dependency, the related works and the framework of our model. In section "Methodology", we transform the traffic prediction into a formula temporal-spatial graph prediction problem. And we describe the framework of SLTTCN, and analyze the composition respectively. In section "Experiment", we conduct the experiments of our model on real world traffic states data and make comparation with the other state-of-the-arts. In section "Conclusion", we make conclusions about our paper and look forward to our further work.

## Methodology

In this section, firstly we give the mathematical definition of problem we analyze in this paper. Secondly, we introduce the two main blocks of our framework, spatial transformer network and the temporal convolution network. Lastly, we summarize the structure of our framework.

### Problem definition and notations

A traffic network can be regarded as a graph $G = (V,E,A)$, where the $V = \{ v_{1} ,v_{2} ,v_{3} ,...,v_{n} \}$ is the collection of all vertices representing the sensors, and the $E = \{ e_{i,j} \}$ is the collection of all edges representing the connectivity among these sensors. The adjacent matrix $A = \{ a_{{ij}} \} \in \mathbb{R}^{{d_{N} \times d_{N} }}$ is the representing the connectivity where the $A_{i,j} = 1$ if $e_{i,j}$ exists otherwise $A_{i,j} = 0$.The traffic prediction is a temporal-spatial problem. The aim of this problem is to use the $X_{F} = \{ x_{{t - T + 1}} ,x_{{t - T + 2}} , \ldots ,x_{t} \} \in \mathbb{R}^{{d_{N} \times d_{T} }}$ which is the input features over the past *T* time slices of traffic state at the time *t* from *N* sensor, to predict the future traffic states $\hat{Y} = \{ x_{{t + 1}} ,x_{{t + 2}} , \ldots ,x_{{t + T}} \} \in \mathbb{R}^{{d_{N} \times d_{T} }}$, it can be formulated as follows:

$$(X_{F} ,G)\mathop{\longrightarrow}\limits^{F}\hat{Y}$$

where the ${\mathcal{F}}$ is the mapping relationship, that is, the model we need to learn.

### Spatial linear transformer network

**Figure 2:**
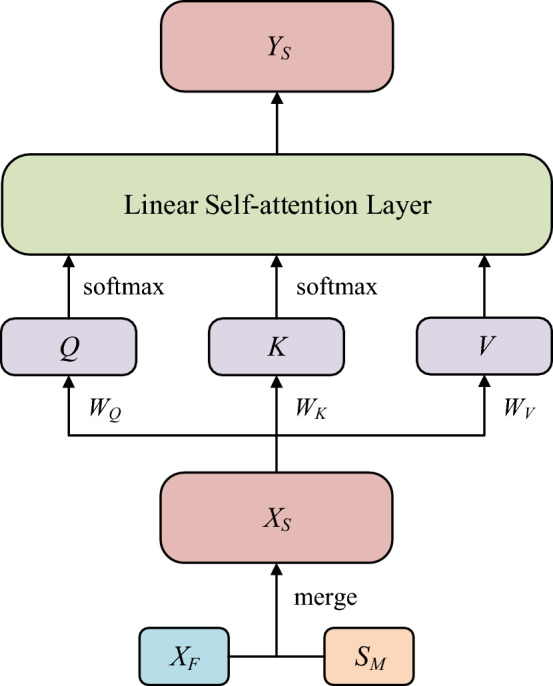
The structure of spatial linear transformer, where the all notations are concretely shown in later part of paper.

In this section, we propose the spatial linear transformer network, which is composed of two parts: the position embedding and the linear self-attention layer. The position embedding is used to incorporate the position information into each node information. The linear self-attention layer is used to capture dynamical spatial dependencies evolving with the time goes by. The structure is shown in Fig. 2.

### Position embedding

Given the original input information $X_{F} \in \mathbb{R}^{{d_{N} \times d_{F} }}$, the spatial-transformer only apply feed-forward operation ignoring the spatial position information of each node. In fact, the position information occupies an import place in modeling spatial correlation. Therefore, what we should do first is to obtain the position embedding $S_{M} \in \mathbb{R}^{{d_{N} \times d_{F} }}$ and merge it into the original input information $X_{F}$, that is $X_{S} = X_{F} + S_{M}$.

**Figure 3:**
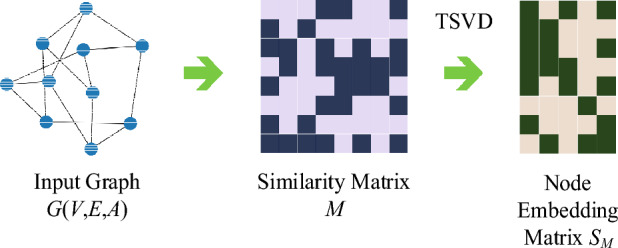
the process of ProNE, where we firstly obtain the similarity matrix *M* from initial graph information, and secondly apply the truncated singular value decomposition (TSVD) to *M* to get the node embedding matrix* S*_*M*_.

The graph embedding theory ProNE^22^ can help us to get the embedding of each node to capture the spatial properties of the graph, and the process is shown in Fig. 3.

We define structure-edge-to which can represent a node-context pair. The edge set then forms a node-context pair set ${{\mathcal D }}=E$.Given node $v_{i}$,the $\overrightarrow {c} _{j} ,\overrightarrow {v} _{i} \in \mathbb{R}^{{d_{F} }}$ respectively represent the embedding of context node $v_{j}$ and embedding of current node $v_{i}$,furthermore the inner product ${\vec{\text{c}}}_{j}^{T} {\vec{\text{v}}}_{i}$ represents the similarity of embedding between context $v_{j}$ and node $v_{i}$.Then we define the occurrence probability matrix $P \in {\mathbb{R}}^{{d_{N} \times d_{N} }}$ where the occurrence probability of context $v_{j}$ is $\hat{p}_{{i,j}} = \sigma \left( {\overrightarrow {c} _{j}^{T} \overrightarrow {v} _{i} } \right)$,where the $\sigma ( \cdot )$ is the sigmoid function.

Here the context only considers directly connected vertices, the loss function is as follows:

1 $$L = - \sum\limits_{{(i,j) \in {{\mathcal D}}}} {p_{i,j} } \cdot \ln \hat{p}_{i,j} = - \sum\limits_{{(i,j) \in {{\mathcal D}}}} {p_{i,j} \cdot } \ln \sigma ({\vec{\text{c}}}_{j}^{T} {\vec{\text{v}}}_{i} )$$

The object function can be expressed as sum of log loss over all edges, where $p_{i,j} = a_{i,j} /d_{i,i}$ and $d_{i,i} = \sum\limits_{j} {a_{i,j} }$.The $p_{i,j}$ represents the normalized weight of $(v_{i} ,v_{j} )$ which is also the regarded as the edge $e_{ij}$ normalized in D.

There is a trivial solution ($\overrightarrow {c} _{j} = \overrightarrow {v} _{i}$ and $\hat{p}_{i,j} = 1$) for the above loss function. The trivial solution means that there only exist positive edges but no negative edges, that is, the edge exists between every node which is unreasonable. For each observed pair of vertices $(v_{i} ,v_{j} )$, the context $v_{j}$ is also may come from negative samples $P_{{{\varvec{\mathcal D}},j}}$,so we update the loss function as:

2 $$L = - \sum\limits_{{(i,j) \in {\varvec{\mathcal D}}}} {\left[ {p_{i,j} \ln \sigma ({\vec{\text{c}}}_{j}^{T} {\vec{\text{v}}}_{i} ){ + }\lambda P_{{{\varvec{\mathcal D}},j}} ( - {\vec{\text{c}}}_{j}^{T} {\vec{\text{v}}}_{i} )} \right]}$$

where $\lambda$ is the negative sample ratio and the negative sample $P_{{{\varvec{D}},j}}$ with context node $v_{j}$ from set of node-context pair can be define as:

3 $$P_{{{\varvec{\mathcal D}},j}} \propto \left( {\sum\limits_{{i:(i,j) \in {\varvec{\mathcal D}}}} {p_{i,j} } } \right)^{\alpha } = \left( {\sum\limits_{{i:(i,j) \in {\varvec{\mathcal D}}}} {\frac{{a_{i,j} }}{{d_{i,i} }}} } \right)^{\alpha }$$

where $\alpha$ is equals to 1 or 0.75^23^.

To minimize the loss function, the sufficient condition is that let its partial derivative with grad to ${\text{c}}_{j}^{T} {\text{v}}_{i}$ equal to zero. Therefore, by calculate $\frac{\partial L}{{\partial ({\text{c}}_{j}^{T} {\text{v}}_{i} )}}{ = }0$, we can get:

4 $$\begin{array}{*{20}c} {{\text{c}}_{j}^{T} {\text{v}}_{i} = \ln p_{i,j} - \ln (\lambda \cdot P_{{{\varvec{D}},j}} )} & {(i,j) \in {\varvec{\mathcal D}}} \\ \end{array}$$

Therefore, we further define a new similarity matrix $M \in \mathbb{R}^{{d_{N} \times d_{N} }}$,where the element of this matrix is:

5 $$M_{i,j} = \left\{ {\begin{array}{*{20}l} {\ln p_{i,j} - \ln (\lambda \cdot P_{{{\varvec{\mathcal D}},j}} )} \hfill & {,(i,j) \in {\varvec{\mathcal D}}} \hfill \\ 0 \hfill & {,(i,j) \notin {\varvec{\mathcal D}}} \hfill \\ \end{array} } \right.$$

Hence, the problem of distributional similarity-based network embedding is transformed to matrix factorization. We use the truncated singular value decomposition (TSVD), that is,$M \approx U_{M} \sum_{M} V_{M}^{T}$, Note that the $\Sigma_{M}$ is the diagonal matrix composed of the largest $d_{F}$ singular values, and the singular values are arranged in descending order, the $U_{M} \in {\mathbb{R}}^{{d_{N} \times d_{F} }}$ and $V_{M} \in {\mathbb{R}}^{{d_{N} \times d_{F} }}$ orthogonal matrices of which column vectors are orthogonal and the eigenvectors corresponding to the selected singular values. Finally, our embedding matrix $S_{M} \in \mathbb{R}^{{d_{N} \times d_{F} }}$ can be expressed as $S_{M} { = }U_{M} \sum_{M}^{1/2}$ where each row in $S_{M}$ represents the embedding of corresponding node.

### Linear self-attention layer

The linear self-attention layer is used to calculate, for every position, an average of the features of all other positions with a weight which is proportional to the similarity score $S_{S} \in \mathbb{R}^{{{ \ominus }d_{N} \times d_{N} }}$ between these features. Formally, the input sequence $X_{S} \in \mathbb{R}^{{d_{N} \times d_{F} }}$ containing position embedding information is projected into three feature matrices, that are called query $Q \in \mathbb{R}{ \ominus }^{{d_{N} \times d_{K} }}$, key $K \in \mathbb{R}{ \ominus }^{{d_{N} \times d_{K} }}$ and value $V \in \mathbb{R}^{{d_{N} \times d_{V} }}$, and the three computed as follows:

6 $$\begin{array}{*{20}c} {Q = X_{S} W_{Q} } \\ {K = X_{S} W_{K} } \\ {V = X_{S} W_{V} } \\ \end{array}$$

where $W_{Q} \in \mathbb{R}^{{d_{F} \times d_{K} }}$, $W_{K} \in \mathbb{R}^{{d_{F} \times d_{K} }}$, $W_{V} \in \mathbb{R}^{{d_{F} \times d_{V} }}$ are the learnable projection matrixes.

Like computing the similarity between node embedding and context embedding, the similarity score $S_{S} \in \mathbb{R}^{{d_{N} \times d_{N} }}$ computed as equation is also the inner product of *Q* and *K* after the softmax function respectively.

7 $$S_{S} = Q{}_{S}K_{S} = {\text{softmax}}(Q){\text{softmax}}(K^{T} )$$

Concretely, the $S_{S(i,j)}$ which is the *i-*th row and *j*-th column element of $S_{S}$ is as follows:

8 $$S_{S(i,j)} { = }\frac{{\sum\limits_{j = 1}^{{d_{K} }} {\exp (Q_{i,j} ) \cdot \exp (K_{i,j} )} }}{{\sum\limits_{j = 1}^{{d_{K} }} {\exp (Q_{i,j} ) \cdot \sum\limits_{i = 1}^{{d_{N} }} {\exp (K_{i,j} )} } }}$$

The softmax function is the exponential normalization function to make *Q* and *K*^*T*^ non-negative and row- normalized, and the multiplication of them $S_{S}$ is also non-negative and row-normalized. Then the output feature $Y_{S} \in \mathbb{R}^{{d_{N} \times d_{T} }}$ of self-attention layer marked as ${\text{Attention}}(Q,K,V)$ is calculated as follows:

9 $$Y_{S} {\text{ = Attention(}}Q{,}K{,}V{) = }S_{S} V_{S}$$

**Figure 4:**
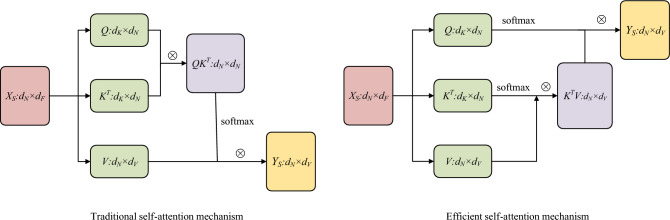
The comparison of Traditional Self-Mechanism and Efficient Self-Attention Mechanism, where the denotes the matrix multiplication.

To reduce the memory and computation resource demand, we adopt the linear self-attention mechanism which a kind of efficient self-attention mechanism^24^. As is shown in picture, because we apply the softmax function for *Q* and *K* respectively, we can switch the computation order which is more efficient. In detail, as is shown in Fig. 4, we firstly compute ${\text{softmax}}\left( {K^{T} } \right)V \in \mathbb{R}^{{d_{K} \times d_{V} }}$ and secondly compute ${\text{softmax(}}Q{)}\left( {{\text{softmax(}}K^{T} {)}V} \right)$, the memory complexity is $O(d_{N} \cdot d_{K} { + }d_{K}^{2} )$ and the computational complexity is $O(d_{N} \cdot d_{V}^{2} )$. Hence the linear self-attention mechanism is efficient in reducing resource requirement by change the computation order when $d_{K} \ll d_{N}$.

To stabilize the training process and prevent overfitting, we further adopt the multi-heads attention mechanism. Specifically, rather than performs a single-head attention operation which computes query, key and value once, the multi-heads attention parallelly projects the *X*_*S*_ to the queries, keys and values for *n*_A_ times of single-head attention operations, then concatenates these outputs and projects again to get the final result. The multi-heads attention H is computed as follows:

10 $${\text{H}} = {\text{Concat}} ({\text{H}}_{1} ,{\text{H}}_{2} ,...,{\text{H}}_{{n_{{\text{A}}} }} )W_{C}$$

with the *i*-th head H_*i*_ computed as:

11 $${\text{H}}_{i} {\text{ = Attention}}(Q_{i} ,K_{i} ,V_{i} )$$

where the $H_{i} \in \mathbb{R}^{{d_{F} \times d_{k} }}$, $W_{C} \in \mathbb{R}^{{(n_{A} \cdot d_{v} ) \times d_{V} }}$ with $d_{K} = n_{{\text{A}}} \cdot d_{k}$ and $d_{V} = n_{{\text{A}}} \cdot d_{v}$.

### Bidirectional temporal convolution network

**Figure 5:**
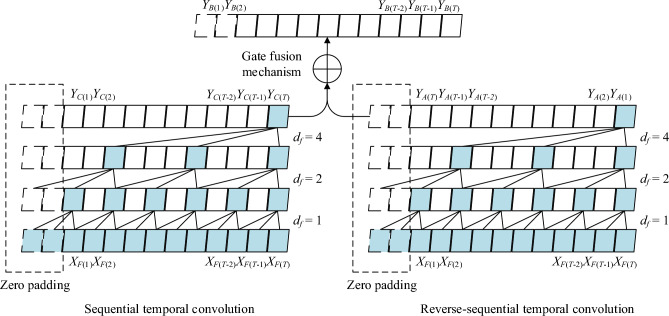
The Bi-TCN layer which is composed of sequential temporal convolution and reverse-sequential temporal convolution layers with the convolution kernel size 3 and the exponential growth of dilation factor *d*_*f*_.

The bidirectional temporal convolution layer is composed of sequential and reverse-sequential temporal convolution layers, which is concretely shown in Fig. 5, and both of the two contain three convolution operations, that are, the one-dimension full convolution, the casual and anti-casual convolution, and the dilated convolution. More specifically, we define the input features $X_{F} \in \mathbb{R}^{{d_{N} \times d_{T} }}$, where the *i*-th element $X_{{F(i)}} \in \mathbb{R}^{{d_{T} }}$ is one dimension sequence which contains *T* time steps. We define the fitter collection $F_{C} \epsilon {\mathbb{R}}^{{d_{N} \times d_{C} }}$, where the *j*-th filter $F_{{C(j)}} \in \mathbb{R}^{{d_{C} }}$ has the kernel size *d*_*C*_. Therefore, the casual convolution output $Y_{C} \in \mathbb{R}^{{d_{N} \times d_{T} }}$ and anti-casual convolution output $Y_{A} \in \mathbb{R}^{{d_{N} \times d_{T} }}$ with $Y_{{C(i)}} \in \mathbb{R}^{{d_{N} }}$ and anti-casual output $y_{{A(i)}} \in \mathbb{R}^{{d_{N} }}$ at *i*-th time step *t* can be formulated as follows:

12 $$\begin{aligned} Y_{C(i)} = & \sum\limits_{j = 0}^{{d_{C} - 1}} {F_{C(j)} \cdot X_{{F(t - d_{f} \cdot j)}} } \\ Y_{A(i)} = & \sum\limits_{j = 0}^{{d_{C} - 1}} {F_{C(j)} \cdot X_{{F(t + d_{f} \cdot j)}} } \\ \end{aligned}$$

where the $d_{f}$ is the dilation factor.

The dilated convolution sampling the input features in fixed interval, and $d_{f}$ controls the ratio of sampling. To capture long-term dependency in exponential growth, we stack the dilation convolution layers with the $d_{f}$ in ascending order.

After we getting the output features, the casual convolution output $Y_{C} \in \mathbb{R}^{{d_{N} \times d_{T} }}$ and anti-casual convolution output $Y_{A} \in \mathbb{R}^{{d_{N} \times d_{T} }}$ of bidirectional temporal convolution layers, we adopt the gate fusion mechanism to fuse them. The Gate fusion mechanism is formulated as follows:

13 $$Y_{B} = \mathcal{G} \odot {\text{Re}} {\text{LU}}(Y_{C} ) + (1 - \mathcal{G}) \odot {\text{Re}} {\text{LU}}(Y_{A} )$$

with the gate $\mathcal{G}$ computed as:

14 $$\mathcal{G} = \sigma (W_{TC} Y_{TC} + W_{TA} Y_{TA} + b_{\mathcal{G}} )$$

where $W_{TC} \in {\mathbb{R}}^{{d_{N} \times d_{N} }}$ and $W_{TF} \in {\mathbb{R}}^{{d_{N} \times d_{N} }}$ are weight matrices, $b_{\mathcal{G}} \in {\mathbb{R}}^{{d_{T} }}$ is the bias. $\sigma ( \cdot )$ is the sigmoid function.${\text{Re}} {\text{LU}}( \cdot )$ is the rectified linear unit as the activation function, is the element-wise product operation. The gate $\mathcal{G}$ fuses the different kinds of input information and control the ratio of them.

### Model structure

**Figure 6:**
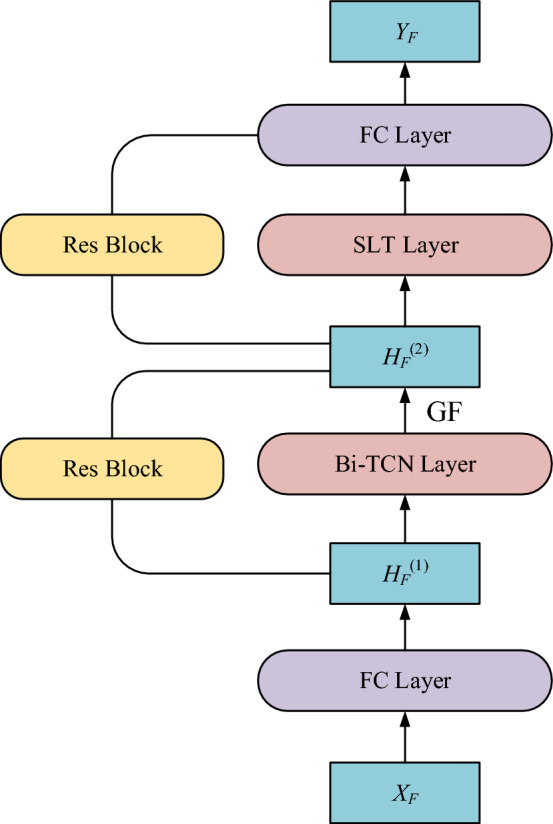
The structure of SLTTCN.

The model structure of SLTTCN are shown in Fig. 6. It consists of spatial linear transformer block (SLT), bidirectional temporal convolution block (Bi-TCN) with gate fusion mechanism (GF), the residual block (Res). we adopt the fully connected (FC) layer firstly transformed the input historical traffic data $X_{F} \in {\mathbb{R}}^{{d_{N} \times d_{T} }}$ to hidden state $H_{F}^{(1)} \in {\mathbb{R}}^{{d_{N} \times d_{F} }}$ and lastly transformed back to the prediction of traffic volume data $Y_{P} \in {\mathbb{R}}^{{d_{N} \times d_{T} }}$. The reason is that the hidden state $H_{F}^{(2)} \in {\mathbb{R}}^{{d_{N} \times d_{{_{F} }} }}$ in spatial linear transformer block adopts the multi-heads attention mechanism, which requires the condition that the dimension $d_{F}$ must be divisible by the number of heads *n*_A_. We adopt the residual block^27^ for stable and efficiently training. We adopt the mean absolute error (MAE) as the loss function to train the model which can be formulated as:

15 $$L(\theta_{L} ) = \frac{1}{T}\sum\limits_{i = t + 1}^{t + T} {\left| {Y_{Pi} - \hat{Y}_{i} } \right|}$$

where the $\theta_{L}$ denotes the all of the parameters in our model. The $Y_{Pi}$ and $\hat{Y}_{i}$ respectively denotes the prediction and ground truth at *i*-th time step.

## Experiment

### Datasets

We conducted experiments on two highway data set to validate our model: the PeMSD4 and PeMSD8. The traffic flow data processing methods we adopt from^25^. Firstly, we exclude some redundant detectors to guarantee the distance between any two adjacent detectors is longer than 3.5 miles. Secondly, we aggregate the raw traffic data every 5 min, which means the traffic data contains 288 timestamps per day. Then, we use the linear interpolation method to fill the missing data and apply the zero-mean normalization operation $x^{\prime} = x - mean(x)$ to the traffic data t the average equals to zero. Last, we split the 60% data for training, 20% data for validation and 20% for testing.

### Metrics

We adopt mean absolute error (MAE), root mean squared error (RMSE) to evaluate our model performance. Note that $Y_{P} = \{ y_{P1} ,y_{P2} , \ldots ,y_{PQ} \} \in {\mathbb{R}}^{{d_{Q} }}$ represents the prediction data, $\hat{Y} = \{ \hat{y}_{1} ,\hat{y}_{2} , \ldots ,\hat{y}_{Q} \} \in {\mathbb{R}}^{{d_{Q} }}$ represents the ground truth, where the *Q*_*N*_ is the size of data set, the MAE and RMSE is calculated as:

16 $${\text{MAE}} = \frac{1}{{Q_{N} }}\sum\limits_{i = 1}^{{Q_{N} }} {\left| {Y_{Pi} - \hat{Y}_{i} } \right|}$$

17 $${\text{RMSE}} = \sqrt {\frac{1}{{Q_{N} }}\sum\limits_{i = 1}^{{Q_{N} }} {\left( {Y_{Pi} - \hat{Y}_{i} } \right)^{2} } }$$

### Baselines

We adopt these baselines as follows to compare with our models: Historical Average model(HA); STGCN^26^; DCRNN^9^; ASTGCN^7^;Graph Wave Net^27^; MTGNN^28^; AGCRN^29^ and PDFormer^30^.

### Settings

For HA model, we adopt the average value of the last 12 time steps to forecast the next time step value. The specific parameters of other models are set according to the original references. For our model, We set the input and output dimensions of each attention head in the Spatial Linear Transformer Network to 12, and the number of attention heads to 4. For the Bidirectional Temporal Convolution Network, we set the input and output dimensions of each layer to the number of nodes, the number of layers, and the convolution kernel size to 3.

### Prediction performance comparison with baselines

We compare the performance of our model and baselines within one hour prediction which contains 12 time slices. The results of 3, 6 and 12 horizon on PeMSD4 and PeMSD8 are shown in Table 1. We can obviously observe from the results that for traditional HA model, its performance is always not ideal because they have the limitation on modeling the traffic data with the complex and nonlinear characteristics. In addition, the other deep learning models outperform the traditional models due to the ability of modeling the nonlinear data.

**Table 1:** The result of different methods on PeMSD4 and PeMSD8.

Model	Dataset	PeMSD4						PeMSD8					
	Metrics	MAE			RMSE			MAE			RMSE		
	Horizon	3	6	12	3	6	12	3	6	12	3	6	12
HA		37.71	37.71	37.71	58.03	58.03	58.03	34.86	34.86	34.86	51.98	51.98	51.98
STGCN		23.43	25.23	31.05	37.42	39.73	46.12	18.92	21.36	26.77	29.35	32.65	37.54
DCRNN		21.33	24.12	30.07	35.11	36.91	42.97	18.31	19.54	23.42	25.97	29.43	35.73
GWNet		20.89	23.64	29.83	33.43	34.34	40.87	17.53	18.12	21.33	25.01	26.03	29.43
MTGNN		19.36	21.21	27.13	30.71	33.07	40.01	17.09	18.43	20.01	23.49	26.01	28.43
ASTGCN		20.83	22.59	26.97	31.51	33.04	39.87	16.75	18.09	20.99	25.57	28.52	32.93
AGCRN		20.24	21.77	26.82	29.43	31.07	37.85	16.39	17.49	19.95	24.73	27.56	31.84
PDFormer		19.44	21.11	26.51	28.53	29.98	37.23	16.03	16.94	19.27	23.63	26.92	29.53
SLTTCN (ours)		**19.21**	**20.65**	**25.11**	**28.02**	**29.61**	**35.21**	**15.21**	**16.74**	**18.58**	**23.45**	**25.87**	**28.22**

Highlight the optimal performance of each horizon in bold.

As shown in Table 1, our method achieves better performance on most metrics on all datasets. This is because, compared to GNN models(STGCN; DCRNN; ASTGCN; GWNet; MTGNN; AGCRN and PDFormer), our proposed Space Linear Transformer Network (SLT) uses self-attention mechanism to calculate the attention weights of each node. Specifically, the weight of self-looping is determined by the feature vector itself, while the weight of interconnection is calculated by the attention score. To balance the weights between self-looping and interconnection, we introduce position encoding in SLT to incorporate distance information between nodes into the calculation of attention weights, thereby capturing spatial relationships between different nodes. The Spatial Linear Transformer network (SLT) can consider the relationships between nodes from a global perspective, rather than just local neighboring nodes. Therefore, SLT is able to better capture long-range dependencies, even when the distance between two nodes is large, it can establish effective connections. In addition, the bidirectional TCN we employed excels in modeling temporal dependencies. The prediction pattern in Bi-TCN (Bidirectional Temporal Convolutional Network) belongs to direct multi-step prediction, avoiding the risk of accuracy degradation that conventional methods may encounter as the time steps increase during the recursive process.

In our Spatial Linear Transformer network, we introduce spatial positional encoding to differentiate the positional information of different nodes. This allows our model to simultaneously consider the similarity between node features and the distance information between nodes. In contrast, traditional GNN models often rely only on neighboring node information and have weaker handling of positional relationships between nodes. Lastly, SLT has a linear structure that enables parameter sharing. This means that the number of parameters to be trained is reduced, which improves computational efficiency of the model.

In subsequent chapters, we also demonstrate the advantages of SLTTCN in terms of computational costs.

### Ablation study

To evaluate the effectiveness of each part in SLTTCN, we conduct ablation studies with 3 variants of our model as follows:

**·w/o** SLT. It removes spatial linear transformer block (SLT).
**·w/o** TCN. It removes bidirectional temporal convolution block (Bi-TCN) with gate fusion mechanism.
**·w/o** Res. It removes residual block (Res).

**Figure 7:**
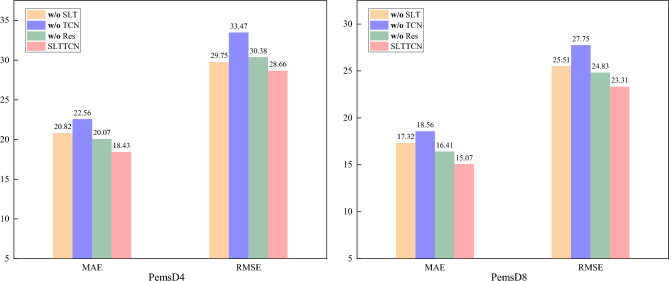
Ablation Study on PEMSD4 and PEMSD8.

Figure 7 reveals the importance of each module for our model performance. The SLT and Bi-TCN modules can respectively model the temporal and spatial dependencies, which are crucial for traffic modeling. Additionally, the performance degradation observed when removing the Res module indicates that the residual connections we adopted effectively alleviate the problem of gradient vanishing and enhance the network's capability to learn nonlinear functions.

### Model analysis

**Figure 8:**
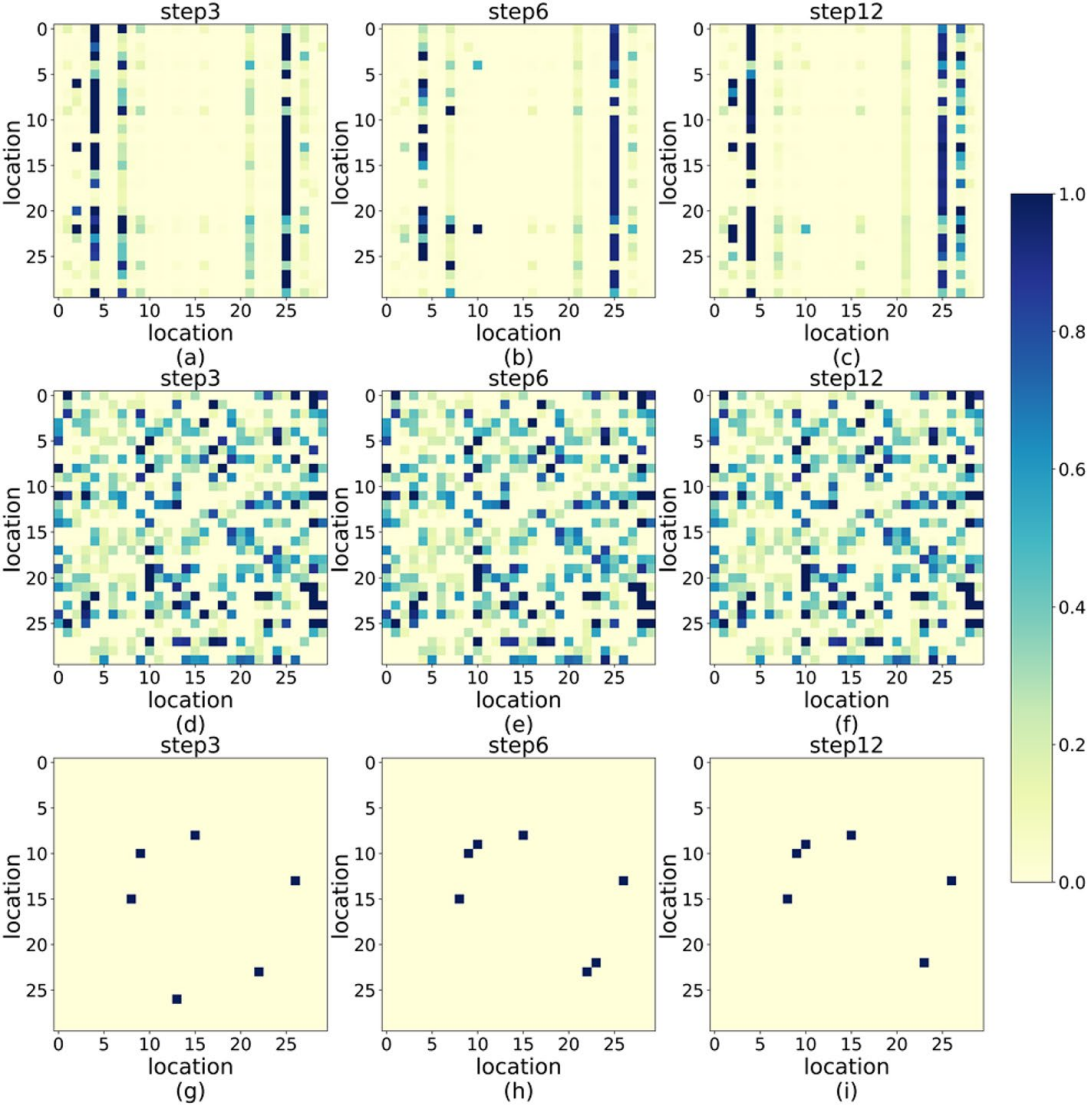
We take first 30 nodes for example in 3rd, 6th and 12th time step on PeMSD4. (**a**, **b**, **c**) Illustration of GDSD. (**d**, **e**, **f**) Illustration of GSSD. (g)(h)(i) Illustration of LDSD.

Firstly, In order to better prove the significance of global dynamic spatial dependency (GDSD) mechanism of SLTTCN, we compare it with other two attention-based spatial models, the one is the local dynamic spatial dependency (LDSD) mechanism which is adopted by GAT, the other is the global static spatial dependency (GSSD) mechanism which is proposed by Graph WaveNet. Their performances are also shown in Table 2. We conclude that firstly, the GDSD and GSSD outperforms the LDSD due to the global dependency which can gather non-local node information, and the GDSD outperform more than LDSD since its dependency is evolving with time. And to observe the three dependency performances, we visualize the three dependencies of first 30 nodes on PeMSD4 data set, and the result is shown in Fig. 8. It is obviously that for GDSD, the spatial dependency distributes on each node and change following the time. For GSSD, the spatial dependency also distributes on each node but fixed. For LDSD, the spatial dependency is distributed on partial nodes and sparse, the reason is that the spatial dependency is computed between the source node and directly connected object nodes, not indirectly connected.

**Table 2:** The comparison of different spatial dependency on PeMSD4.

Model	15 min		30 min		60 min	
	MAE	RMSE	MAE	RMSE	MAE	RMSE
SLTTCN (GDSD-TCN)	18.43	29.66	19.42	31.38	21.05	33.88
GLSD-TCN	18.94	29.93	19.72	31.55	21.52	34.26
GSSD-TCN	18.56	29.86	19.69	31.45	21.39	34.07

Secondly, to demonstrate the computational efficiency of our novel attention mechanism-based SLTN and the fused gate mechanism-based Bi-TCN, we compared the time costs of the main baseline models with SLTTCN on the PemsD4 dataset. We record the average consuming time for each epoch of training and the total consuming time of validating. The result is presented in Table 3, we can see that HA is the fastest because of its simplest structure among all models. We can observe that STGCN is efficient during training due to its fully convolution structure during the training process, and the GWNet is slower due to its combination of diffusion graph convolution and self-adaptive adjacency matrix. DCRNN adopts an iterative approach instead of directly generating all predictions, which increases training time. Meanwhile, AGCRN has a significantly increased number of parameters to learn node-specific patterns, which slows down the computation speed. The ASTGCN is slower because it combing the spectral graph convolution operation and attention mechanism, and the running time is also impacted by the stack of spatial–temporal blocks. The delay module of Pdformer significantly increases the computational cost of the model. Thanks to the linear structure of the spatial transformer and the parallel computation of TCN, our model is the most efficient.

**Table 3:** The comparison computation time on PeMSD4 dataset.

Model	Computation Time	
	Training (s/epoch)	Inference (s)
HA	7.76	0.14
STGCN	16.47	0.63
DCRNN	39.69	3.48
GWNet	37.66	2.31
ASTGCN	47.22	10.03
AGCRN	34.67	2.65
PDFormer	121.83	18.02
SLTTCN	12.07	0.26

## Conclusion

In this paper, we propose a novel deep learning model for traffic flow forecasting called SLTTCN. It can capture the spatial dependency by spatial linear transformer which belongs to dynamical graph convolution operation including the graph embedding theory for learning embedding of each nodes. It also capture the time dependency by bidirectional temporal convolution which can construct the time relationship among muti-step data from past to future and vice versa. The performance and computational efficiency of SLTTCN on PeMSD4 and PeMSD8 data set outperforms all the baselines we chose. Furthermore, we will explore more methods for traffic forecasting. For data set and information, there are more external factors which also influence the traffic flow, such as weather, social events and traffic policy and so on. For model structure, we consider mining the edges relationship of traffic network and combing with the nodes features domain in our future study.

## Data Availability

The data that support the findings of this study are openly available at https://pems.dot.ca.gov/.
